# Preventive Effects of Dairy Products on Dementia and the Underlying Mechanisms

**DOI:** 10.3390/ijms19071927

**Published:** 2018-06-30

**Authors:** Yasuhisa Ano, Hiroyuki Nakayama

**Affiliations:** 1 Research Laboratories for Health Science & Food Technologies, Kirin Company Ltd., 1-13-5 Fukuura Kanazawa-ku, Yokohama-shi, Kanagawa 236-0004, Japan; 2 Laboratory of Veterinary Pathology, Graduate School of Agricultural and Life Sciences, the University of Tokyo, 1-1-1 Yayoi, Bunkyo-ku, Tokyo 113-8657, Japan; anakaya@mail.ecc.u-tokyo.ac.jp

**Keywords:** amyloid β, Alzheimer’s disease, cognitive function, dairy products, dementia, inflammation, microglia

## Abstract

Alongside the rapid population aging occurring worldwide, the prevention of age-related memory decline and dementia has become a high priority. Dairy products have many physiological effects owing to their contents of lactic acid bacteria and the fatty acids and peptides generated during their fermentation. In particular, several recent studies have elucidated the effects of fermented dairy products on cognitive function. Epidemiological and clinical evidence has indicated that fermented dairy products have preventive effects against dementia, including Alzheimer’s disease. Recent preclinical studies have identified individual molecules generated during fermentation that are responsible for those preventive effects. Oleamide and dehydroergosterol have been identified as the agents responsible for reducing microglial inflammatory responses and neurotoxicity. In this review, the protective effects of fermented dairy products and their components on cognitive function, the mechanisms underlying those effects, and the prospects for their future clinical development will be discussed.

## 1. Introduction

With the rapid aging of the population worldwide, cognitive decline and dementia are becoming increasing burdens not only on patients and their families, but also on national healthcare systems. Dementia is a general term for memory loss and other mental abilities severe enough to interfere with daily life. It is caused by physical changes in the brain. The most common type of dementia is Alzheimer’s disease, which comprises 50–70% of dementia cases. Alzheimer’s disease is histopathologically characterized by the presence of amyloid beta (Aβ) plaques and intracellular neurofibrillary tangles (NFTs) consisting of hyperphosphorylated tau. Both Aβ senile plaques and NFTs are formed of insoluble, densely-packed protein filaments. The accumulation of Aβ plaques and NFTs correlates with the symptoms of Alzheimer’s disease and results in neuronal damage and death [1,2,3,4]. Other common types of dementia include vascular dementia [5], Lewy body dementia [6], and front temporal dementia [7]. Numerous reports have demonstrated that the formation of Aβ plaques is followed by inflammation in the brain, which is closely associated with NFTs and accelerates the development and progression of Alzheimer’s disease [3,4,8,9]. The mechanisms that regulate Aβ plaques, NFTs, and inflammation are important targets for the therapy and prevention of Alzheimer’s disease. However, owing to the lack of a disease-modifying therapy for dementia, preventive approaches such as diet, exercise, and active learning are being explored. In addition, high blood pressure, smoking, diabetes, and obesity are risk factors for dementia [10]. A recent study suggested that stress is also a risk factor for Alzheimer’s disease [11]. Therefore, it is important to reduce these risk factors through adopting appropriate lifestyle habits.

There is now substantial evidence that dietary factors can modify the risk of dementia. Based on the results of several epidemiological investigations, the Mediterranean diet has been receiving increasing attention as a nutritional approach for lowering the risks of dementia [12,13,14]. In addition, specific dietary components including docosahexaenoic acid (DHA) from fish oil [15,16,17,18], resveratrol from red grapes [19,20,21], and curcumin from turmeric [22,23,24] have been evaluated for their potential protective effects against dementia or cognitive decline in several clinical trials. Recent epidemiological and clinical studies have indicated that fermented dairy products and their components, including lactic acid bacteria as well as peptides and fatty acids generated during fermentation, may also protect against dementia or cognitive decline. In this review, the recent studies investigating the effects of the intake of fermented dairy products on the risks of dementia and the underlying mechanisms demonstrated by recent studies will be discussed.

## 2. Epidemiological Studies on the Relationship between Fermented Dairy Product Consumption and Cognitive Function

Recent epidemiological studies have suggested that the consumption of dairy products, including yogurt and low-fat cheese, may reduce the risk of cognitive decline in the elderly and contribute to the prevention of Alzheimer’s disease. Camfield et al. [25] suggested in their review that specific components of dairy products including bioactive peptides, colostrinin, proline-rich polypeptides, α-lactalbumin, vitamin B12, calcium, and probiotics might promote healthy brain function during aging. They also suggested that low-fat dairy products, when consumed regularly as part of a balanced diet, may have several beneficial outcomes for neurocognitive health during aging. However, the underlying mechanisms that benefit cognitive function have not been elucidated.

Crichton et al. [26,27] revealed that individuals who consumed low-fat dairy products, including yogurt and cheese, once a week had a higher cognitive function than those who did not. Previous reports had suggested that low-fat dairy products might lower the risks of obesity [28,29,30], type 2 diabetes [31,32], and cardiovascular diseases, which are all linked with the risks of cognitive decline and dementia [33,34,35]. They evaluated pre-existing data from 1183 participants and examined associations between dairy intake and self-reported cognitive and memory functions, self-esteem, stress, anxiety, mood, and psychological well-being. Dairy intake was calculated using a self-completed, quantified food frequency questionnaire (FFQ) and detailed information regarding the intakes of milk, cheese, ice cream, cream, yogurt, and dairy desserts as well as the fat content of each item was analyzed from the FFQ. The results revealed that the intakes of dairy and macronutrients were significantly associated with psychological health measures. In men, a higher protein intake was associated with lower perceived stress scores. Analyses of individual dairy foods found that the consumption of low-fat yogurt was positively associated with memory performance (quality of recall) in men. In women, the consumption of low-fat cheese was positively associated with social functioning and negatively associated with perceived stress levels. The intake of cheese was reported to be associated with decreased cognitive impairment in a population of late middle-aged to elderly people [36]. These results suggested that low-fat yogurt and cheese are beneficial for cognitive functions.

Ozawa et al. [37] surveyed more than 1000 Japanese subjects who were living in a local community, aged 60–79 years, and free from dementia to investigate any potential association between their diet and their risks of dementia. Their dietary patterns were surveyed using a 70-item semi-quantitative FFQ, and their average daily nutritional intakes were calculated from the weekly frequency and portion size of various foods. Their health status was monitored by several methods including a neuropsychological test. Dietary patterns associated with the risk of dementia were assessed using a reduced rank regression analysis [38]. This analysis extracted seven dietary patterns that explained 87.1% of the total variation in the intakes of the following seven nutrients, which were selected as responsible variables: saturated fatty acid, monounsaturated fatty acid, polyunsaturated fatty acid, vitamin C, potassium, calcium, and magnesium. Seven dietary factors were associated with a preventive effect against the risks of dementia that was greater than 20% in magnitude. These protective factors were soybeans and soybean products, green vegetables, other vegetables, algae, and milk and dairy products. In contrast, a high intake of rice was associated with an increase in the risks of dementia greater than 20% in magnitude. Thus, a dietary pattern characterized by high intakes of soybeans and soybean products, vegetables, algae, and milk or dairy products together with a low intake of rice was associated with a reduced risk of dementia. These results support the contention that a high intake of milk and dairy products helps to prevent cognitive decline.

The subsequent research of Ozawa et al. [39] focused on the effects of milk and dairy intake on the development of all-cause dementia and its subtypes in an elderly Japanese population. During 17 years of follow-up on individuals aged 60 years or older who did not initially have dementia (*N* = 1081), 303 subjects developed all-cause dementia. The age- and sex-adjusted incidences of all-cause dementia, Alzheimer’s disease, and vascular dementia all showed a significant inverse correlation with the intake of milk and dairy products. After adjusting for potential confounders, the linear inverse relationship between the intake of milk and dairy products and the development of Alzheimer’s disease remained significant, whereas the relationships with all-cause dementia and vascular dementia were not significant. The investigation showed that a greater intake of milk and dairy products was associated with a reduced risk of dementia, especially Alzheimer’s disease, in the general Japanese population.

## 3. Clinical Trials for the Improvement of Cognitive Function by Dairy Products

In a clinical trial, Ogata et al. [40] found that the intake of dairy products was strongly associated with better short-term memory. The association was significant both with and without adjustment for genetic and family environment factors using a sample of twin pairs. Short-term memory was evaluated using the “logical memory I” (LM-I) scores from the Japanese version of the revised Wechsler memory scale. Participants were asked to listen to two short stories and immediately recall the details. The final analysis was performed using data from 78 men and 278 women. All individual-level analyses using generalized estimating equations showed that dairy product intake was significantly associated with the LM-I scores in men. In addition, a within-pair analysis using within-monozygotic and within–dizygotic pair-difference scores showed a significant association between the intake of dairy products and LM-I scores in men. Furthermore, a within-pair analysis using within-monozygotic pair-difference scores indicated a significant association between the intake of dairy products and LM-I scores in men. Among men, a high intake of dairy products was significantly associated with better short-term memory after adjustment for possible covariates. The authors concluded that the intake of dairy products may prevent cognitive decline regardless of genetic and family environment factors in men.

Markus et al. [41,42] demonstrated that the intakes of α-lactalbumin-rich whey protein isolate improved cognitive performance in stress-vulnerable subjects. They evaluated the effects of the intake of tryptophan-rich whey protein on the ratio of plasma tryptophan to the sum of the other large neutral amino acids (Trp-LNAA ratio) and cognitive performance in high stress-vulnerable subjects. Their double-blind, placebo-controlled, crossover study included 29 high stress-vulnerable subjects and 29 low stress-vulnerable subjects. A significantly greater increase in the plasma Trp-LNAA ratio was observed after the consumption of the α-lactalbumin-rich diet than after the consumption of the control diet. Cognitive performance was evaluated using a computerized Sternberg memory scanning task, and the subjects’ reaction time and amount of errors across the different subtasks were measured. The mean reaction time showed a significant difference between the high and low stress-vulnerable subjects. Furthermore, the reaction time of the high-stress-vulnerable subjects was significantly lower after consuming the α-lactalbumin diet (758 ± 137 ms) than it was after consuming the control diet (800 ± 173 ms). An increase in the plasma Trp-LNAA ratio is considered to be an indirect indicator of increased brain serotonin function, which results in the improvement of cognitive performance. The authors suggested that the intake of an α-lactalbumin-rich diet increases the level of tryptophan and serotonin in the brain and improves cognitive performance in stress-vulnerable subjects.

## 4. Preventive Effects of Dairy Products Fermented with *Penicillium candidum* against the Pathology of Alzheimer’s Disease

Recent epidemiological and clinical studies have suggested that a high intake of dairy products may have preventive effects against cognitive decline and Alzheimer’s disease. Following this finding, it is important to elucidate the mechanism and responsible molecular components. Using transgenic model mice, Ano et al. [43] demonstrated that the intake of Camembert cheese, which is a fermented dairy product, displayed preventive effects against Alzheimer’s disease. In the experiment, 5xFAD mice were used as an Alzheimer’s disease model. These mice overexpress mutant human amyloid precursor protein with the Swedish (K670N, M671L), Florida (I716V), and London (V717I) familial Alzheimer's disease mutations along with human presenilin 1 harboring two familial Alzheimer’s disease mutations, namely M146L and L286V. The 5xFAD mice display Aβ depositions, plaques, and severe inflammation in the brain in addition to impaired cognitive function. 5xFAD mice aged 3–6 months were given food with or without an extract from Camembert cheese. The brain tissues of mice fed with the Camembert cheese extract showed reduced levels of inflammatory cytokines including tumor necrosis factor α (TNF-α) and macrophage inflammatory protein 1α. The amounts of Aβ_1-42_ quantified by enzyme-linked immunosorbent assay (ELISA) and detected immunohistochemically were reduced in the group fed with the Camembert cheese extract. The abundances of the brain-derived and glial-derived neurotropic factors increased. These results showed that certain components of Camembert cheese contribute to the suppression of the inflammation and reduction of Aβ in the brain. In subsequent studies of the same research group, the responsible agents in Camembert cheese that contribute to the prevention of Alzheimer’s disease pathology, especially inflammation in the brain, were explored.

## 5. Neuronal Inflammation Accelerates the Pathology of Alzheimer’s Disease

Several reports have demonstrated that inflammation in the brain following the formation of Aβ plaques and NFTs is closely associated with the development and progression of Alzheimer’s disease [3,8,9]. Inflammation in the brain is mainly regulated by microglia, which remove apoptotic cells and waste products such as Aβ through phagocytosis and also contribute to the host defense against virus infection in the central nervous system [44,45]. Microglia play an important role in clearing Aβ to regulate the pathology of Alzheimer’s disease [46]. Recent studies revealed the crucial roles of microglia in maintaining the brain environment and cognitive function. However, in the brain tissues of patients with Alzheimer’s disease, microglia infiltrate around the Aβ plaques, become excessively activated, and chronically produce inflammatory mediators such as TNF-α, macrophage inflammatory protein 1α, reactive oxygen species, and nitric oxide. These inflammatory mediators are toxic to neurons and cause neuronal cell death subsequent to Aβ deposition [47]. Under normal physiological conditions, microglia are important for the maintenance of brain environment; however, under pathological conditions, microglia can become excessively activated and negatively contribute to the brain environment.

Numerous reports have suggested that controlling the activities of microglia may contribute to the prevention and cure of Alzheimer’s disease and cognitive decline. Epidemiological studies have suggested that the prolonged use of nonsteroidal anti-inflammatory drugs, including a common medication, ibuprofen, significantly reduces the risk of developing Alzheimer’s disease [48,49,50,51]. Consistent with those results, a long-term ibuprofen treatment was found to significantly suppress microglial inflammation and the development of Aβ plaques in a transgenic mouse model of Alzheimer’s disease [52,53]. 

Before Ano et al. evaluated the effects of a Camembert cheese extract using 5xFAD transgenic Alzheimer’s model mice, they assessed the effects of various fermented dairy products on microglia [43]. Their results indicated that dairy products fermented with the fungi *Penicillium candidum* and *Penicillium roqueforti* suppressed the inflammatory responses of primary microglia. Their findings suggested that *Penicillium* fermentation is essential for the anti-inflammatory activity, although other dairy products that were unfermented or fermented did not suppress the microglial inflammatory responses. The intake of Camembert cheese extract reduced inflammatory responses and Aβ production in the hippocampus of 5xFAD transgenic mice, while it increased the production of the brain-derived and glial-derived neurotropic factors. Various fatty acids and peptides were also generated during the fermentation of dairy products with the *Penicillium* fungi. Ano et al., in addition, investigated the molecular components of Camembert cheese that are responsible for preventing the development of Alzheimer’s disease pathology.

## 6. Effects of Oleamide and Dehydroergosterol Generated during the Fermentation of Dairy Products with *Penicillium* Fungi on Brain Inflammation

**Figure 1:**
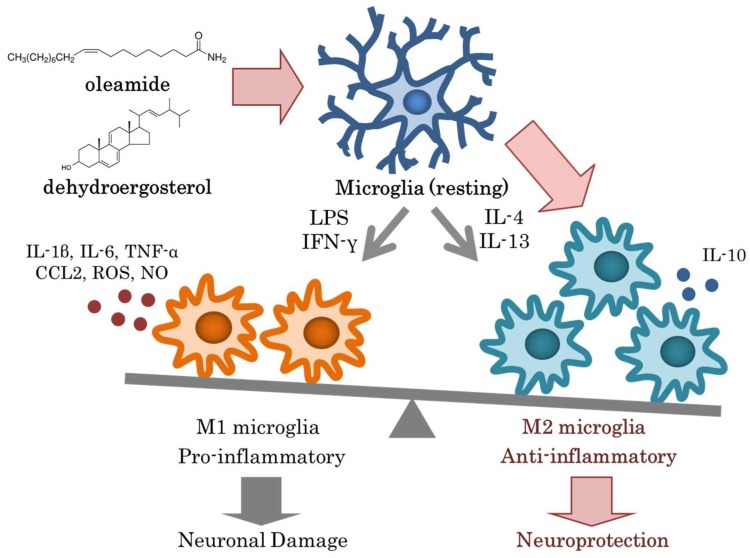
Modulation of microglial activation by dairy products.

Ano et al. [43,54] identified oleamide and dehydroergosterol as two components of Camembert cheese that were responsible for suppressing microglial inflammation. Oleamide is an amide of oleic acid [55,56] (Figure 1) that forms naturally in the body of animals and might be synthesized during the fermentation of dairy products by *Penicillium* fungi [57]. Oleamide accumulates in the cerebrospinal fluid during sleep deprivation and induces sleep in animals; thus, it has potential applications in the treatment of mood and sleep disorders [58,59]. Oleamide not only suppresses the production of inflammatory cytokines and chemokines but also increases the microglial phagocytosis of Aβ. In addition, it induces the differentiation of microglia into the anti-inflammatory M2 type. A recent study using murine macrophages showed that oleamide suppressed the induction of iNOS and COX-2 by lipopolysaccharides (LPS) via preventing the nuclear translocation of NF-κβ by suppressing the phosphorylation of Iκβ-α [60]. Oleamide is also known as an endogenous substance that binds to cannabinoid receptor 2 (CB2), which is mainly expressed on the surface of immune cells (monocytes, macrophages, and B cells). CB2 is also expressed on microglia and contributes to the inhibition of microglia-mediated neurotoxicity by reducing the production of pro-inflammatory molecules [61]. In addition, CB2 activity facilitates the transformation of microglial cells from the M1 to M2 phenotype, which is suggested to favor phagocytosis and reparative mechanisms [62]. Several studies have proposed a direct role for CB2 in the modulation of Aβ peptide levels in the brain. Most of those studies have suggested that CB2 participates in Aβ clearance rather than in Aβ production and aggregation. In the case of amyloid precursor protein/presenilin 1 mice lacking CB2, the increased Aβ deposition observed may be related to a reduced phagocytotic activity of microglia in their brains [63], considering the role of CB2 activity in promoting microglial-induced Aβ phagocytosis [64,65]. These reports support the contention that oleamide, which is an agonist of CB2, suppresses the microglial inflammation and enhances the phagocytosis of Aβ, resulting in a preventive action against Alzheimer’s disease. CB2 has been receiving increasing attention as a therapeutic target for Alzheimer’s disease [66,67,68]. Ano et al. discovered that the concentration of oleamide in dairy products depends on the type of dairy product and the fermentation process used. Therefore, the dietary intake of oleamide can be increased via supplementation or the consumption of specific dairy products. 

Dehydroergosterol was also discovered as a component of Camembert cheese that suppressed microglial inflammation. Dehydroergosterol is an analogue of ergosterol, which is a sterol found in the cell membrane of fungi [69,70] (Figure 1). Dehydroergosterol is generated by fungi during fermentation. Dehydroergosterol suppresses the LPS-induced inflammatory response (including TNF-α production) of primary microglia in a concentration-dependent manner, whereas ergosterol does not display this activity. Microglia treated with dehydroergosterol were observed to differentiate into the anti-inflammatory M2 phenotype. The number of apoptotic neurons detected by staining for caspase 3/7 decreased after co-culturing the cells with a microglial culture supernatant treated with dehydroergosterol and LPS as compared with an LPS-only control. These results suggested that dehydroergosterol suppresses the inflammatory responses of microglia and exerts a neuroprotective effect via promoting synaptic extension and neuronal survival [71]. The amount of dehydroergosterol produced during fermentation varies among different *Penicillium* strains, so it might be possible to increase the amount of dehydroergosterol in dairy products by optimizing the fermentation processes.

The mechanisms by which oleamide and dehydroergosterol regulate inflammation in the brain have been receiving increasing attention, because neural inflammation is involved not only in dementia but also in other neuronal disorders [72,73] including depression [74,75], anxiety disorder [76], and chronic fatigue [77]. A recent study demonstrated that the intakes of oleamide shows antidepressant-like effects in mice subjected to the forced swimming test via the activation of cannabinoid receptors [78]. In another demonstration, oleamide improved schizophrenia-like symptoms in mice treated with an NMDA receptor antagonist, MK-801 [79]. The balance of microglial differentiation between M1 pro-inflammatory type and M2 anti-inflammatory type is important to maintain homeostasis in the central nervous system. Oleamide has the potential to have preventive effects against the development of several brain disorders. Daily habits including nutrition, sleep, and exercise are closely related to the maintenance of the microglial balance and the prevention of neuronal disorders. Future studies will further elucidate the effects of active ingredients of fermented dairy products against depression and other brain diseases.

## 7. Conclusions

This review introduced recent advances regarding the protective effects of dairy product intake against dementia and cognitive decline. The reports regarding these issues will help with the development of new approaches for the prevention of dementia. Oleamide and dehydroergosterol were discussed in this review as responsible agents for these protective effects of dairy products (Figure 1). However, the functions of the other various fatty acids and peptides generated during fermentation have not yet been elucidated. Future studies are expected to elucidate the mechanisms underlying the physiological benefits of dairy products in more detail. Based on the current evidence, the regular intake of dairy products and their molecular or microbial components seems to have the potential to contribute to the prevention of dementia and cognitive decline.

Oleamide and dehydroergosterol identified from Camembert cheese induce microglia into the M2 anti-inflammatory phenotype, leading to neuroprotection. The mechanisms that regulate microglial activation and inflammation in Alzheimer’s disease are important targets for disease prevention. The regulation of microglia via daily lifestyle habits has been receiving increasing attention. The intake of neuroprotective and anti-inflammatory compounds including oleamide and dehydroergosterol in meals is safe and easy, so nutritional approaches are promising for the prevention of neurodegenerative disorders. 

## Abbreviations

Aβ	Amyloid β
CB2	Cannabinoid receptor 2
FAD	Familial Alzheimer’s disease
FFQ	Food frequency questionnaire
LM-I	Logical memory I
LPS	Lipopolysaccharides
NFT	Neurofibrillary tangle
TNF-α	Tumor necrosis factor α
